# Announcement: 10th International Symposium on Trace Elements in Man and Animals, TEMA 10

**DOI:** 10.1155/MBD.1997.291

**Published:** 1997


					10TH INTERNATIONAL SYMPOSIUM
ON TRACE ELEMENTS IN MAN AND ANIMALS
TEMA 10
May 2-7, 1999, Congress Hall, Evian, France
The 10th birthday of TEMA will be held on the south bank of Lake Leman at the base of the
snowcapped Alps, in Evian, French hydromineral and floral touristic resort. The TEMA symposium
series represents 30 years of research in the field of Trace Elements. The series originated in
Aberdeen, UK in 1969. Symposia have been held in Madison, USA, 1973; Freising, Germany,
1977; Perth, Australia, 1981; Aberdeen, UK, 1984; Pacific Grove, USA, 1987; Dubrovnik, Croatia,
1990; Dresden, Germany. 1993 and Banff, Canada, 1996. The TEMA Symposia aim is to provide a
forum to discuss recent progress on the functional roles and metabolism of trace elements among
workers investigating functional aspects of the biology of trace elements and those whose tasks are
to apply their findings in the fields of nutrition, human and veterinary medicine, agriculture, ecology
and environmental sciences.
Registration and submission of abstracts
Contributed papers will be welcome. Instructions for registration and submission of abstracts will be
detailed in the Second Circular, available in January 1998.
Accomodations
Rooms at special symposium rates will be reserved at several hotels in four different price categories
near the Congress Hall and in a Holiday Residence. For students, accomodation in a residential
complex will be arranged at reduced prices.
Presentations
Sessions will include plenary and parallel papers oral and poster presentations as well as workshops
of the conference topics. Poster and oral presentations will be considered equally important.
Official Language
The official language of the symposium will be English
Publication
All conference manuscripts will be peer reviewed and accepted contributions will be published in
full or as 3-7 page contribution by a major International Publisher after the symposium.
Topics
Bioavallablty of trace elements.
Intra-cellular metabolism of metals (transporters channels storage and distribution).
Change in the trace element body distribution under conditions of deficiency and toxicity.
New functional role for trace elements.
Newer trace elements.
Oxidative stress and trace elements.
The paradox of iron: from essential to toxic.
Trace elements and gene regulation.
Trace elements and apoptosis.
Trace element-related inherited diseases.
Trace elements in exercise.
Trace elements in aging.
Trace elements in diabetes.
Trace elements in pregnancy lactation and pediatrics.
Trace elements in inflammation and immunity.
Trace elements in cardiovascular diseases.
Trace elements in intensive care.
Trace elements in dermatology.
Animal trace element needs and deficiencies.
Trace elements in pet animal diets and diseases.
291
Trace elements in livestock.
Assessment of trace element consumption in human.
Recommended trace element dietary allowance.
Policy and safety of trace element supplementation.
Trace elements and the water supply.
Epidemlologlcal and Intervention trials.
Therapeutic uses of trace elements.
Occupational and environmental exposure.
New indicators of trace element status.
New analytical methods: ICP-MS,HPLC, hybride-ICP.
Speciation of trace elements in the body.
Trace element intracellular determination.
Trace element quality control organisation and accreditation.
Preliminary program of the workshops: Chromium, Iron, Trace elements and the skin, Trace element
supplementation in elderly.
Social Program
The planned social program includes a welcome party prior to the opening of the Congress, an
afternoon visit of the Nestle Research Center in Lausanne (30 minutes by boat) with a dining cruise
on the lake and the Congress banquet at the Evian Casino. Post conference sight-seeing
excursions in Burgundia and French Riviera will be arranged
TEIViA 10 Chairman:
A. E. Favier, Universit Joseph Fourier, Grenoble, France
International Parent Committee
M. Anke, Germany
J. R. Arthur, UK, Treasurer
I. Bremner, UK, Chairman
N. D. Costa, Australia
P. W. F. Fisher, Canada
R. Gibson, New Zealan
J. King, USA
B. Mocilovic, USA
J. R. Prohaska, USA
R. A. Sunde, USA
Advisory Scientific Committee
R. A. Anderson, USA
Po Braetter, Germany
P. Chappuis, France
J. Neve, Belgium
Y. Rayssiguier, France
Local Organizing Committee
A. M.RousseI,Treasurer and A. Alcaraz ,Secretary
University Hospital of Grenoble, France
For further informations,please contact:
A. Alcaraz
Laboratoire de Biochimie C, Hopital Albert Michallon, BP 217,
F-38043 Grenoble cedex 9, France
Tel: 33 4 76 76 54 84 Fax: 33 4 76 76 56 64
e-mail" Anne-Marie.Roussel @ ujf-grenoble.fr
or Arlette.Alcaraz @ ujf-grenoble.fr
or
U RL" http :llwww-sante.ujf-grenoble.frlPHARMAItemaI 0/tema. htm
292

				

